# Marked hypereosinophilia in a toddler: a case report

**Published:** 2011-02-25

**Authors:** RM Stoicescu, CM Mihai, AD Giannakopoulou

**Affiliations:** * Ovidius University, Faculty of Pharmacy, ConstantaRomania; ** Ovidius University, Faculty of Medicine, ConstantaRomania; *** Microbiology Department, Psychiatric Hospital of ThessalonikiGreece

**Keywords:** zoonosis, geophagia, pets exposure

## Abstract

**Background.** Human toxocariasis is primarily a soil–transmitted zoonosis, so children with geophagia are at an increased risk of toxocariasis, especially those living in homes with puppies that have not been dewormed.

**Case report**. A 17–months–old female presented to our department with fever, abdominal distention and marked eosinophilia. Iron deficiency anemia, marked leukocytosis (79,000 cells/mm^3^) accompanied by  marked eosinophilia (55,000 cells/mm^3^), and hyper–gammaglobulinemia were noted. On the basis of the strong serological positivity for toxocariasis, marked eosinophilia, and low–density lesions in the liver at computed tomography, a diagnosis of visceral larva migrans syndrome was made.

**Conclusion.** Visceral larva migrans is usually suspected in a young child with history of geophagia,  pets exposure, hepatomegaly, whose complete blood count reveals leukocytosis and marked eosinophilia.

Abbreviations: VLM–visceral larva migrans, OLM–ocular larva migrans, ESR–erythrocyte sedimentation rate, HBV– hepatitis B virus, HCV–hepatits C virus, EBV–Epstein–Barr virus, CMV– cytomegalovirus infection, HIV–human immunodeficiency virus, CT– computed tomography, ELISA–Enzyme–linked immunosorbent assay, CBC–complete blood count.

## Introduction

Human toxocariasis is a helminthozoonosis due to the infection of humans by ascarid larvae belonging to the genus Toxocara. For many years this helminthiasis was regarded as an uncommon pediatric disease.

The availability of sensitive and specific immunodiagnostic tests has greatly improved our knowledge on toxocariasis. Humans normally become infected by ingestion of embryonated eggs (each containing a fully developed larva, L2) from contaminated sources. Humans are accidental hosts, which inhibits L2 larvae maturation within humans [1].  There are three main syndromes: visceral larva migrans (VLM), which encompasses diseases associated with major organs; covert toxocariasis, which is a milder version of VLM; and ocular larva migrans (OLM), in which pathological effects on the host are restricted to the eye and the optic nerve [2]. 

The first description was made in the early 1950's by H.C. Wilder, when he published a paper describing ocular granulomas in patients thought to have retinoblastomas. Two years later, Beaver et al. published the presence of *Toxocara larvae* in granulomas removed from patients with symptoms similar to those in Wilder's patients [3].

Human toxocariasis is primarily a soil–transmitted zoonosis. Geophagia or soil eating is a specific type of pica that increases the risk of toxocariasis, especially in children living in homes with puppies that have not been dewormed. Poor personal hygiene as well as consumption of raw vegetables grown in contaminated kitchen gardens may result in chronic low–dose infections [4].

Physiological reactions to Toxocara infection depend on the host's immune response and the parasitic load [5]. Most cases of Toxocara infection are asymptomatic, especially in adults [6]. When symptoms do occur, they are the result of migration of second stage Toxocara larvae through the body.

High parasitic loads or repeated infection can lead to visceral larva migrans (VLM) [6]. VLM is primarily diagnosed in young children, because they are more prone to exposure and ingestion of infective eggs. Toxocara infection commonly resolves itself within weeks, but chronic eosinophilia may result [7].  In VLM, larvae migration incites inflammation of internal organs and sometimes the central nervous system. Symptoms depend on the organ(s) affected [8]. 

## Case Report

**Figure 1:**
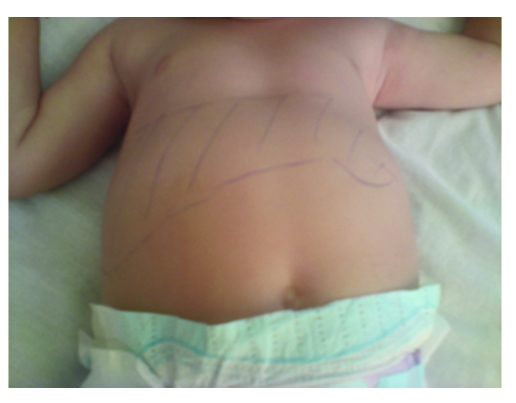
Abdominal distention. Hepatosplenomegaly

A 17–months–old female presented to the Pediatric Department from Constanta County Hospital with fever, abdominal distention and marked eosinophilia. The patient was coming from a rural area. Her medical history was clear and she was well, according to her mother, until 1 week before admission, when she developed palor, fever, abdominal pain, and abdominal distention.  The patient came with a strong history of geophagia, and exposure to puppies at home.  The acute signs included: abdominal pain, decreased appetite, restlessness, and fever. Upon admission, physical examination revealed mild pallor of the skin and conjunctivae, and abdominal distention. There was no lymphadenopathy, but hepatomegaly and modest splenomegaly were noted (Figure 1); the body temperature was 38.2ŶC.

**Figure 2:**
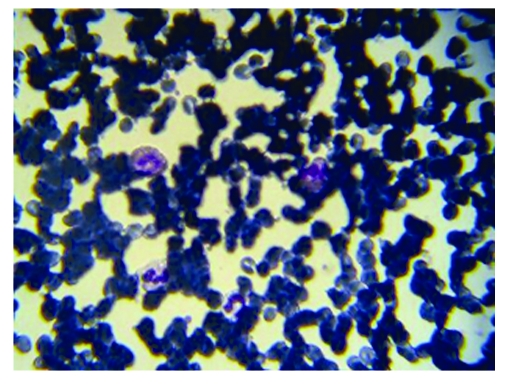
Hypereosinophilia. Peripheral smear

Iron deficiency anemia (Hemoglobin 7.3 g/dl, serum iron level 23 ug/dl), marked leukocytosis (79,000 cells/mm3) accompanied by  marked eosinophilia (55,000 cells/mm3) (Figure 2), platelet count 445 x 106/l, ESR 10 mm/1 h, and hyper–gammaglobulinemia (IgM, IgG, and IgE classes)  were noted.

Her sister, 4–y–o, had a strong history of pica, recurrent wheezing, and epilepsy. The family is from an isolated village, living under extremely unsanitary conditions and in dire poverty.

Multiple stool, blood and urine cultures were negative. There was no serological evidence for a recent viral infection from HBV, HCV, EBV, CMV, or HIV. Serological tests were also negative for *Salmonella* infections, trichinellosis, echinococcosis and cysticercosis, but were strongly positive for toxocariasis. Enzyme2013;linked immunosorbent assay for the detection of IgG specific toxocaral antibodies using secretory–excretory antigen showed 2.5 U (>1.1). For the evaluation of the results index positivity was used (absorbance of each tested sample divided by the mean absorbance of the cut–off was obtained in the sample test); the cut–off was calculated and appointed to 0.3. According to the manufacturer recommandations, an index positivity >1.1 was considered positive , 0.9–1.1 as borderline and <0.8 as negative for *Toxocara canis* infection.

**Figure 3:**
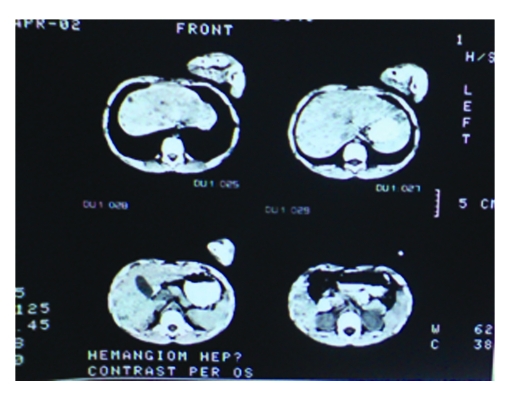
Computed tomography showing multiple liver low–density lesions

Computed tomography scans of the lungs and brain were unremarkable, but multiple liver nodules were detected as low-density lesions on computed tomography (Figure 3).

**Figure 4:**
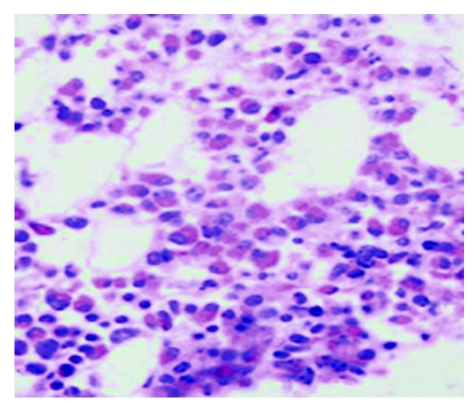
Bone marrow biopsy, with 50% to 60% eosinophils

A bone marrow aspirate and biopsy revealed increased cell infiltration of the bone marrow with the predominance of eosinophils (Figure 4).

Ultrasound of the heart and ophthalmoscopy were normal. On the basis of the strong serological positivity for toxocariasis and the marked eosinophilia, diagnosis of VLM syndrome was made. Primary hypereosinophilic syndrome has been ruled out in this patient, based on her clinical picture and laboratory testing. Idiopathic hypereosinophilic syndrome is characterized by prolonged eosinophilia without an identifiable underlying cause and multiple–organ dysfunction, most frequently involving the heart, the central or peripheral nervous system and the lungs.

The patient received a 10-day course of albendazole (200 mg orally twice daily) and demonstrated significant clinical improvement. 

**Figure 5:**
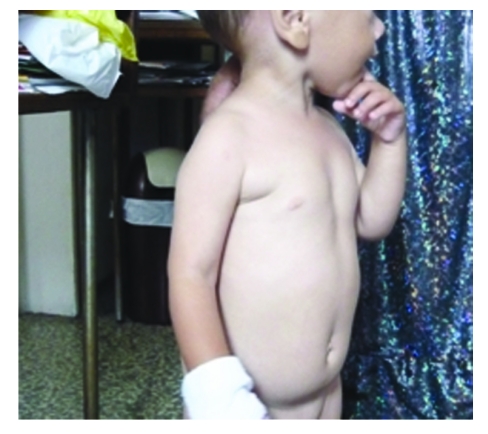
Normal abdominal size without hepatosplenomegaly after 1 year.

After appropriate treatment the number of eosinophils decreased, but one year after initial diagnosis, she continues to have eosinophilia, despite the treatment with albendazole. Hepatosplenomegaly and abdominal distention regressed to normal (Figure 5).

*Toxocara canis* eggs were found in the dirt surrounding the house . These eggs were most probably the common source of infection. 

**Figure 6:**
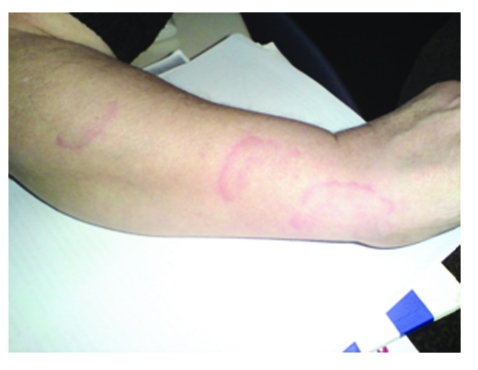
Grandmother's forearm. The subcutaneous migration tracks of the larvae

The subcutaneous migration tracks of the larvae were seen in patient's grandmother (Figure 6).

Both grandmother and patient’s sister have a positive ELISA test for Toxocara canis, without eosinophilia.

## Discussion

Transmission of *Toxocara* to humans is usually through ingestion of infective eggs. These eggs are passed in cat or dog feces, but the defecation habits of dogs cause T. canis transmission to be more common than that of T. cati. Both Toxocara canis and Toxocara cati eggs require a several week incubation period outside of a host before becoming infective, so fresh eggs cannot cause toxocariasis. Widespread environmental contamination with Toxocara eggs, the attraction of children to the animals and their environment, and the play habits of children combine to facilitate human infection with Toxocara spp.

*Toxocara* infections are generally asymptomatic, and the seroprevalence varies from 3% to 86% in different countries [9].

Figueiredo [10] reported that *T.canis* infection must be considered in at–risk children, such as those with puppies at home, who have had contact with soil.

Fan [11] also reported those children who admitted living in a household where dogs were kept or playing in soil appeared at increased risk of seropositivity for Toxocara infection. Iddawela [12] reported that dog ownership, especially puppies, and geophagia–pica, were significant risk factors.

Alderete [9] reported that among infected children, the mean age was 9.4 years, with a similar distribution between genders. A significant association was observed with the presence of onychophagia, residence with a dirty backyard, living in a slum, previous wheezing episodes, school attended, and family income.

Our patient came from a rural area characterized by poverty and her family had a low monthly income. Also, our patient is a toddler with a strong personal history of geophagia–pica, living in a dirty backyard and playing every day with puppies, so, she was the ‘ideal’ candidate for a Toxocara canis infection. More, in her family, there are two persons with toxocara infection:  her sister, 3–y–o, with history of pica, recurrent wheezing, and epilepsy and their grandmother in whom were seen subcutaneous migration tracks of the larvae (Figure 6). Both had positive ELISA for Toxocara canis. None had eosinophilia. 

Our case is typical of an individual severely affected by visceral larval migrans due to the larvae of the Toxocara species. Most cases of hepatic toxocariasis show few or no symptoms, and eosinophilia, often found incidentally, is a common initial clue to suspect helminthiasis [13].

Several studies have evidenced that visceral toxocariasis can be detected by CT; hepatic lesions are seen as low density areas on CT [14, 15].

Hepatic involvement of VLM is common due to portal venous drainage of visceral organs.  In our patient, a clinical history with laboratory findings of marked eosinophilia, a rise in serum total immunoglobulin and a positive serologic test along with computed tomography of the affected organ confirm the diagnosis of toxocariasis.

Visceral larva migrans (VLM) is usually suspected in a young child with geophagia and exposure to pets, with fever and hepatomegaly, whose CBC  reveals leukocytosis and marked eosinophilia.

Infection with Toxocara species is probably far more common than reported, given the large stray dog population and areas of primitive sanitary conditions. ELISA tests could be used to reveal additional infections in Romania.
